# Neuroprotective effect of piracetam-loaded magnetic chitosan nanoparticles against thiacloprid-induced neurotoxicity in albino rats

**DOI:** 10.1007/s10787-023-01151-x

**Published:** 2023-02-06

**Authors:** Mohamed Abomosallam, Basma M. Hendam, Amr A. Abdallah, Rasha Refaat, Ahmed Elshatory, Heba Nageh Gad El Hak

**Affiliations:** 1 Forensic Medicine and Toxicology Department, Faculty of Veterinary Medicine, Mansoura University, Mansoura, 35516 Egypt; 2 Husbandry and Development of Animal Wealth Department, Faculty of Veterinary Medicine, Mansoura University, Mansoura, 35516 Egypt; 3 Central Agricultural Pesticides Laboratory, Agricultural Research Center, Giza, 12619 Egypt; 4 Phytochemistry and Plant Systematics Department, National Research Center, Dokki, Giza, 12622 Egypt; 5 Forensic Medicine and Clinical Toxicology Department, School of Medicine, Cairo University, Cairo, 11865 Egypt; 6 Zoology Department, Faculty of Science, Suez Canal University, Ismailia, Egypt

**Keywords:** Thiacloprid, Piracetam, Magnetic chitosan nanoparticles, Brain, Neurotoxicity

## Abstract

Thiacloprid (TH) is a neurotoxic agricultural insecticide and potential food contaminant. The purpose of this study was to investigate the relationship between TH exposure and memory dysfunction in rats, as well as the potential protective effect of piracetam and piracetam-loaded magnetic chitosan nanoparticles (PMC NPs). Rats were divided into five equal groups (six rats/group). The control group received saline. Group II was treated with PMC NPs at a dose level of 200 mg/kg body weight (Bwt); Group III was treated with 1/10 LD_50_ of TH (65 mg/kg Bwt); Group IV was treated with TH (65 mg/kg Bwt) and piracetam (200 mg/kg Bwt); Group V was co-treated with TH (65 mg/kg Bwt) and PMC NPs (200 mg/kg Bwt). All animal groups were dosed daily for 6 weeks by oral gavage. Footprint analysis, hanging wire test, open field test, and Y-maze test were employed to assess behavioral deficits. Animals were euthanized, and brain tissues were analyzed for oxidative stress biomarkers, proinflammatory cytokines, and gene expression levels of glial fibrillary acidic protein (GFAP), amyloid-beta precursor protein (APP), B-cell lymphoma 2 (Bcl-2), and caspase-3. Brain and sciatic nerve tissues were used for the evaluation of histopathological changes and immunohistochemical expression of tau protein and nuclear factor kappa B (NF-κB), respectively. The results revealed that TH-treated rats suffered from oxidative damage and inflammatory effect on the central and peripheral nerves. The administration of PMC NPs considerably protected against TH-induced neuronal damage, increased antioxidant enzyme activity, decreased inflammatory markers, and improved behavioral performance than the group treated with piracetam. The neuroprotective effect of PMC NPs was mediated through the inhibition of GFAP, APP, caspase-3, Tau, and NF-κB gene expression with induction of Bcl-2 expression. In conclusion, TH could induce oxidative stress, inflammatory and neurobehavior impairment in rats. However, PMC NPs administration markedly mitigated TH-induced brain toxicity, possibly via oxidative and inflammatory modulation rather than using piracetam alone.

## Introduction

Pesticides are chemical compounds used to repel and control unwanted pests such as insects, fungi, weeds, and rodents (Rani et al. 2021). Although pesticide application serves many important agricultural purposes especially crop protection, pesticides are not highly selective and are also toxic to non-target organisms, including mammals, and have been linked with many neurodegenerative diseases (Arab and Mostafalou 2021). Several pesticides, including insecticides, can induce neurotoxicity (Laetz et al. 2020). Neurotoxic insecticides, which decapitate insects by interfering with chemical neurotransmission or ion channels in their nervous system, comprise organophosphates, organochlorines, carbamates, pyrethroids, neonicotinoids, and other compounds (Vikash et al. 2018). Neonicotinoids, the most extensively used insecticide worldwide nowadays, are nicotine derivatives and classified into *N*-nitroguanidines (imidacloprid, thiamethoxam, and dinotefuran) and *N*-cyano-aminides (acetamiprid and TH) (Anadón et al. 2020; Costas-Ferreira and Faro 2021). Neonicotinoids selectively bind to insect nicotinic acetylcholine receptors (nAChRs) in the nervous system and block the alpha subunits of nAChRs, ligand-gated ion channel, causing neuromuscular paralysis and death (Perry et al. 2021; Taillebois et al. 2018). TH, like other neonicotinoids, is highly selective but recent studies have revealed that certain neonicotinoids can cross the blood–brain barrier (BBB) with higher affinity to mammalian nAChRs, like nicotine, and lead to neurotoxicity (Abdallah et al. 2021; Hendawi et al. 2016; Mora-Gutiérrez et al. 2021; Zhang et al. 2021). Furthermore, TH could promote ion channel opening and the influx of sodium (Na^+^) and calcium (Ca^2+^) into the neuronal cells resulting in excitatory overstimulation with cognitive impairment and structural damage at the hippocampal level (Houchat et al. 2020; Mora-Gutiérrez et al. 2021).

Piracetam is a γ-aminobutyric acid (GABA) cyclic derivative and a synthetic nootropic, that can enhance cognitive function besides overcoming oxidative stress and membrane changes accompanying neurodegenerative diseases and prevent its progression without the well-known mechanism of action (Chaturvedi et al. 2021; Nasr and Wahdan 2019). However, recent studies demonstrated that piracetam may interact with the cell and mitochondrial membranes and change the cell membrane's fluidity and function offering neuroprotection against neurodegenerative diseases associated with mitochondrial dysfunction and oxidative stress (Severyukhin et al. 2021; Sheref et al. 2022; Vikash et al. 2018). Since nootropics act on the brain, bypassing the BBB is crucial (Jampilek et al. 2015; Nasr and Wahdan 2019). Despite its critical role, BBB is an impediment when it comes to drug delivery. Thus, new technologies and non-invasive routes are explored recently (Polkovnikova et al. 2017; Teleanu et al. 2018). Polymeric nanoparticles recently attracted a lot of interest as an intriguing delivery system and they can combine various active compounds into their inner cores by physical entrapment or chemical conjugation with relatively elevated therapeutic efficacy and high stability (Ahmed and Badr-Eldin 2020; Wang and Xu 2017). Their uptake into the brain is hypothesized to occur via receptor-mediated endocytosis followed by transcytosis (Kreuter 2014). Chitosan, one of the most abundant biopolymers, is a linear polyamine with free hydroxyl and amine groups (Nagpal et al. 2013a). Furthermore, it can be cross-linked with multivalent anions, mucous membranes, and cell surfaces because of its cationic properties which invoke tight junction and enhance membrane absorption via prolonging the absorption site residual time so chitosan is a suitable choice for the preparation of biodegradable, stable, and biocompatible nanoparticulate nootropics delivery systems (Nagpal et al. 2013b; Yu et al. 2019). The coupling of magnetite to chitosan nanoparticles can improve drug uptake by increasing the surface charge and concentrating it in the target area (Assa et al. 2017; Shevtsov et al. 2018). Therefore, we aimed to prepare PMC NPs as a novel nanocomposite system for enhanced brain uptake. The prepared nanocomposite was characterized and evaluated for its effectiveness against TH-induced neurotoxicity in albino rats after oral administration.

## Materials and methods

### Synthesis of Fe_3_O_4_ nanoparticles

Fe_3_O_4_ nanoparticles were prepared via the chemical co-precipitation method as reported previously by Petcharoen and Sirivat (2012). Briefly, in 100 ml of distilled water, 5.94 g of FeCl_3_ and 3.06 g of FeSO_4_ were combined, and 1 M NaOH was used to adjust the pH to 10. The mixture was then mechanically stirred at 80 °C for 1 h. The prepared magnetite nanoparticles were magnetically separated, repeatedly cleaned with deionized water, then dried in a vacuum drying oven at 60 °C for 12 h.

### Synthesis of modified magnetic chitosan nanoparticles (MC NPs)

MC NPs were synthesized by chitosan cross-linking with sodium tripolyphosphate (TPP) in the presence of magnetite nanoparticles as previously reported by Zhang et al. (2020). 100 mg of Fe_3_O_4_ nanoparticles were combined with 100 ml of acidified chitosan solution (pH 4). Then TPP solution (1 mg/ml) was slowly added with magnetic stirring for 1 h at atmospheric temperature. Afterward, the products were magnetically separated and repeatedly washed with ethanol, vacuum dried at 60 °C for 12 h and stored at 4 °C.

### Synthesis of PMC NPs

Piracetam loading was carried out on the synthesized MC NPs as reported previously with some modifications (Unsoy et al. 2014). The mixtures of MC NPs (8 mg/ml) and piracetam (4 mg/ml) were magnetically stirred (90 rpm) at an atmospheric temperature in phosphate buffer saline (PBS) (pH 6) for 5 h. Then PMC NPs were magnetically separated. The encapsulation efficiency (EE) and loading capacity (LC) of PMC NPs were measured with a UV spectrophotometer at 264 nm after generating a calibration curve. Then calculated by Eqs. (1) and (2), respectively (Taherian et al. 2021).

1 $${\text{EE}}\;\left( {\text{\% }} \right) = \frac{{{\text{Total amount of the drug}} - {\text{free amount of the drug}}}}{{\text{Total amount of the drug}}} \times 100$$

2 $${\text{Loading}}\;{\text{capacity}}\;\left( \% \right) = \frac{{{\text{Total}}\;{\text{amount}}\;{\text{of}}\;{\text{the}}\;{\text{drug}} - {\text{free amount of the drug}}}}{{\text{Weight of nanoparticles in solution}}} \times 100$$

### Characterization of PMC NPs and in vitro drug release

PMC NPs were characterized through morphological examination with the transmission electron microscope (TEM) and Fourier transform infrared spectroscopy (FTIR) prepared in potassium bromide (KBr) as a pellet at a frequency range of 400 to 4000 cm^−1^ to confirm nanoparticles synthesis.

In vitro release profile of piracetam from PMC NPs was analyzed by the dialysis bag method (Eskandani et al. 2022). Briefly, a known amount of PMC NPs corresponding to 20 mg of piracetam was suspended in 2 ml of phosphate-buffered saline (PBS) and placed in a dialysis bag (12 kDa) which was incubated in 200 ml of PBS release medium (pH 6.8, 7.4, and 2, respectively) with stirring (150 rpm) at 25 °C. 1 ml of in vitro medium was withdrawn at predetermined time intervals (1, 2, 4, 6, 8, 12, 16, 20, 24 h) and the amount of piracetam was analyzed spectrophotometrically at 215 nm and a curve was drawn depicting the cumulative drug release percentage at various time intervals.

### Experimental animals

Thirty adult female albino rats (150 ± 10 g) were used in this study. Rats were obtained from the faculty of Veterinary Medicine animal unit, Mansoura University. All normal acclimatization conditions were met as 12 ± 1 h light–dark, 22 ± 2 °C temperature, and feeding on a standard diet with free water access then acclimatized for 2 weeks before the experimental study. All animal-related procedures were conducted according to the animal's rights with the formal approval of the animal's ethical committee of Mansoura University (No R/149, 2022).

### Experimental design

**Fig. 1:**
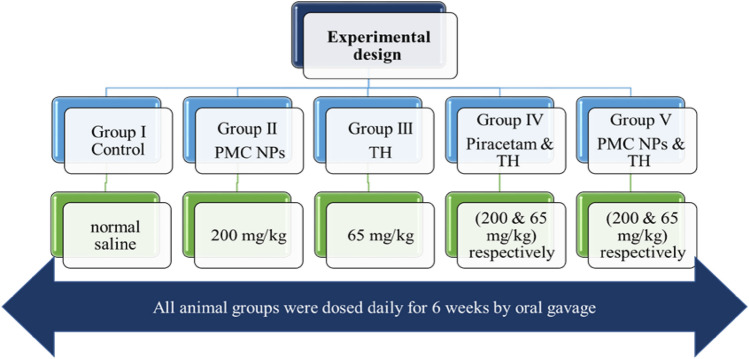
Schematic diagram for the experimental design

Rats were subdivided into five equal groups (six rats per group) (Fig. 1). The control group received 1 ml of normal saline daily. Group II was treated with PMC NPs at the dose of 200 mg/kg Bwt. based on previous reports (Mehta et al. 2014; Trofimov et al. 1993; Zaitone et al. 2012) which revealed that piracetam at a dose level of 200 mg/kg Bwt. could ameliorate peripheral neuropathic pain, motor impairment, and behavioral disturbances, respectively. Group III was treated with 1/10 LD_50_ of TH (65 mg/kg Bwt) which was determined by an up-and-down procedure following organization for economic co-operation and development (OECD) guidelines for acute oral toxicity testing of chemicals (Bruce 1985). Group IV was treated with TH (65 mg/kg Bwt) and piracetam (200 mg/kg Bwt). Finally, Group V was co-treated with TH (65 mg/kg Bwt) and PMC NPs (200 mg/kg Bwt). All animal groups were dosed daily for 6 weeks by oral gavage.

### Assessment of behavioral neurotoxicity

#### Footprint analysis

Footprint patterns were used to predict animal gait (Brooks et al. 2012). In brief, rats were trained to pass straight forward through a narrow path (80 × 15 × 30 cm) furnished with white paper. The animals' fore- and hind-paws were painted red and blue, respectively, and then allowed to pass through the runway. The footprints’ patterns were analyzed for base width, stride length, and paw overlapping (Garabadu and Agrawal 2020).

#### Hanging wire test

Motor coordination and neuromuscular function were assessed by the hanging wire test (Santhanasabapathy et al. 2015). Briefly, experimental rats were suspended by their forelimbs on a stretched wire (90 cm length) and elevated 50 cm from a flat surface. The time (s) until the animal fell was recorded, with a 90-s cut-off time (Nagakannan et al. 2012).

#### Open field test

This test was performed to evaluate anxiety-related behaviors and general locomotor activity (Crépeaux et al. 2017). The test was carried out in a rectangular arena (100 × 80 × 60 cm). The floor was divided into 25 equal squares defined as internal 9 white squares and external 16 red squares. Rats were placed individually at the center of the open field and left free to explore for 5 min. During this period, the time spent in the center and peripheral areas, time of immobility and rearing frequency were recorded (Ghasemnejad-Berenji et al. 2021).

#### Y-maze test

The Y-maze test was performed to evaluate the short-term spatial memory of rodents (Hussein et al. 2018). The Y-maze consisted of three equal arms (70 × 15 × 20 cm), labeled A, B, and C. Each rat was placed at arm A and allowed to move freely through the maze for 8 min and the series of arms entries and alterations were recorded. An arm entry was registered once all four paws of the rat were within the arm completely. The spontaneous alternation behavior, an index of spatial short-term memory, was considered when the animal enters all three arms on consecutive order or alternating triplets (ABC–BAC), but repetitive sequences (ABA–BCC) were eliminated, and the spontaneous alternation behavior percentage was determined as follows: (Farokhcheh et al. 2021)

$$\text{Alternation }({\%}) = \frac{\text{Number of successful alterations }(\text{actual alterations}) }{\text{Total number of arm entries}-2 (\text{possible alterations})}\times 100$$

### Biochemical and histological examination

#### Tissue preparation

At the end of the study, all rats were anesthetized by intraperitoneal injection of thiopental sodium (30 mg/kg Bwt.) and serum separated immediately after drawing blood from the retro-orbital cavity (Barai et al. 2019). Afterward, rats were sacrificed by injecting higher doses of thiopental sodium and the entire brain was dissected from the skull, washed with cold normal saline, then divided into two halves for biochemical, histopathological, and immunohistochemical examination (Ghasemnejad-Berenji et al. 2021). Tissue homogenates were prepared in ice-cold PBS (pH 7.4, 10% or 20% w/v homogenates) and centrifuged at 10,000*g* at 4 °C for 15 min, and then the supernatant obtained was separated into autoclaved vials and stored at − 80 °C until further biochemical assays (Li et al. 2018).

#### Determination of acetylcholinesterase (AChE) activity

The activity of the AChE enzyme in the brain tissue was estimated spectrophotometrically according to the standard Ellman assay (Madiha et al. 2021). Brain homogenate (20%), phosphate buffer (pH 8.0), and 100 μl 5,5′-dithio-bis-nitrobenzoic acid (DTNB) were mixed and the absorbance was measured at 412 nm. The reaction started after the addition of 5 μl of acetylthiocholine (ATC), and the absorbance change was recorded at 0.5 min intervals for 2 min. The AChE activity was determined as nanomoles of substrate consumed per minute per mg tissue.

### Determination of oxidative stress and antioxidant enzyme activities

#### Determination of superoxide dismutase (SOD) activity

SOD activity was evaluated based on nitro blue tetrazolium (NBT) reduction to blue formazan (Beauchamp and Fridovich 1971). Change in absorbance was measured at zero time and after 5 min at 560 nm. The specific activity was determined as units per gram of tissue (Haider et al. 2014).

#### Determination of catalase (CAT) activity

CAT activity was carried out based on Aebi, Mörikofer-Zwez, and von Wartburg (1972) method whereas PBS (50 mM, pH 7.4) and H_2_O_2_ (30 mM) were mixed at 25 °C with tissue homogenate. The decomposition of H_2_O_2_ was monitored at 240 nm spectrophotometrically for 60 S. CAT activity was determined as units/gm tissue.

#### Determination of glutathione S-transferase (GST)

GST activity was carried out according to Ogunsuyi et al. (2021). In brief, tissue homogenate (100 µl), potassium phosphate buffer (70 mM, pH 7.4), chloro-2,4-dinitrobenzene (CDNB), and glutathione (3.20 mM) were mixed at 25 °C. The GST activity was recorded at 340 nm for 10 min and expressed as units/g tissue.

#### Determination of reduced glutathione (GSH) level

GSH content was evaluated according to the method of Moron et al. (1979). Brain homogenate supernatant was mixed with an equal amount of trichloroacetic acid (TCA, 10%) at 25 °C and centrifuged (3000 rpm for 5 min at 25 °C), then the supernatant was collected and added to phosphate buffer (0.1 M, pH 7.4) and DTNB (0.04%). GSH level was measured at 412 nm and expressed as mg/g tissue.

#### Determination of lipid peroxidation biomarker malonaldehyde (MDA) content

MDA content was measured according to Chow and Tappel (1972). Brain homogenate was added to TCA (10%) and thiobarbituric acid (0.375%) and boiled in the water bath (95 °C for 20 min), then cooled and centrifuged (2000 rpm for 15 min). The light pink colored supernatant absorbance was then recorded at 532 nm. Tissue MDA levels were expressed as nmol/g tissue.

#### Assessment of proinflammatory cytokines

Interleukin-6 (IL-6), interleukin 1β (IL-1β), and tumor necrosis factor-alpha (TNF-α) were colorimetrically detected in the brain tissue according to the manufacturers' instructions based on the sandwich and competitive ELISA technique using commercially available kits for rat IL-6 (BMS625), (IL-1β) (BMS630), and TNF-α (BMS622), (Invitrogen, Thermo Fisher Scientific, MI, USA). IL-6, IL-1β, and TNF-α concentrations were measured in brain homogenate as pg/ml at 450 nm by interpolation from a standard curve via a micro-plate ELISA reader (Sorin Biomedica SpA., Milan, Italy) with a detection limit from 31.3–2000 pg/ml, 31.3–2000 pg/ml, and 39.1–2500 pg/ml, respectively (Jiang et al. 2021).

#### Quantitative real-time PCR

To investigate the pathophysiology and molecular mechanism of TH-induced neurotoxicity and the protective role of PMC NPs, GFAP, APP, caspase 3, and Bcl-2 mRNA expression was evaluated by quantitative real-time PCR (qRT-PCR). Total RNA was extracted from frozen brain tissues via the TRIzol method based on the manufacturer’s protocol. First, RNA was extracted following homogenization with TRIzol reagent (Thermo Fisher Scientific, USA, 15596018). The purity and concentration of the isolated RNA were checked through NanoPhotometer^®^ spectrophotometer at 260 and 280 nm absorbance. Second, the first-strand cDNA was synthesized from the extracted RNA by a Quantitect^®^ Reverse Transcription kit (Qiagen, Germany) following the guidelines of manufacture. Then specific forward and reverse primers were used to amplify target genes; their sequences are presented in Table 1 using β-actin as a housekeeping gene (internal control) for normalization of the expression levels of target genes.

**Table 1:** List of primers used in RT-PCR reactions

Target gene	Primers’ sequences	References
*GFAP*	F: 5′-CAGACTTTCTCCAACCTCCAG-′3 R: 5′-CTCCTGCTTCGAGTCCTTAATG-′3	Doorn et al. (2015)
*APP*	F: 5′-TGGGTTGACAAACATCAAGACAGAA-′3 R: 5′-GCACCTTTGTTTGAACCCACATC-′3	Ying-Cai et al. (2007)
*Caspase3*	F: 5′-AGTTGGACCCACCTTGTGAG-′3 R: 5′-AGTCTGCAGCTCCTCCACAT-′3	Ibrahim et al. (2020)
*Bcl2*	F: 5′-CTGGTGGACAACATCGCTCTG-′3 R: 5′-GGTCTGCTGACCTCACTTGTG-′3	Wang et al. (2010)
*Β-actin*	5′-TCCTCCTGAGCGCAAGTACTCT-′3 5′-GCTCAGTAACAGTCCGCCTAGAA-′3	Banni et al. (2010)

The mRNA expressions of GFAP, APP, caspase-3, and BCL-2 were quantified in the brain tissues by qRT-PCR, Rotor-Gene SYBR Green PCR Kit (Qiagen, Germany). The amplification conditions were: 95 °C for 10 min, followed by 40 cycles of amplification (95 °C for 15 s, 60 °C for 20 s, and 70 °C for 20 s.). The expression pattern for each gene was calculated via the comparative 2^− ΔΔ*Ct*^ method as mentioned by Livak and Schmittgen (2001).

#### Histopathological and immunohistochemical examination

Half of the brain samples (cerebrum, cerebellum, and hippocampus) and sciatic nerve were fixed in 10% neutral formalin, dehydrated immediately and embedded in paraffin according to the paraffin-embedding technique (Bancroft and Layton 2012), mounted in blocks, and cut into 5-μm thickness slices using a microtome. Coronal slide sections of the brain were stained with hematoxylin and eosin (H&E) and crystal violet. Coronal slide sections of the sciatic nerve were stained with H&E and silver nitrate. Rat’s brain and sciatic nerve were examined and the different pathologic observed lesions were evaluated by light microscope.

Assessment of immunohistochemical expression of tau protein in the brain's cerebral cortex and cerebellum and NF-κB in sciatic nerve was performed. For tau protein (monoclonal antibody, Cat (A0024), Dako dilution 1: 15,000) and NF-κB (monoclonal antibody Cat (6H7L22), Thermo Scientific, USA dilution 1:20), brain and sciatic sections (4 μm thickness) were deparaffinized, rehydrated followed by antigen retrieval by boiling with citrate buffer (10 mM, pH 6.0) at 105 °C for 10–20 min followed by room-temperature cooling. Endogenous peroxidase was deactivated by H_2_O_2_ (3%) in absolute methanol for 5 min, then brain and sciatic sections were incubated with anti-tau protein for the brain and anti-NF-κB for sciatic nerve antibody at 4 °C overnight. Sections were counterstained with Mayer's hematoxylin solution. Five random fields from each section were captured from original representative micrographs using a digital camera. The percentage of immune reaction was assessed via ImageJ software according to Khafaga et al. (2021).

#### Statistical analysis

Data were presented as mean ± standard deviation (SD) and analyzed by one-way analysis of variance (ANOVA), followed by Tukey post hoc test using GraphPad Prism 5 software. Statistical significance was set at *P* value < 0.01.

## Results

### Characterization of PMC NPs

**Fig. 2:**
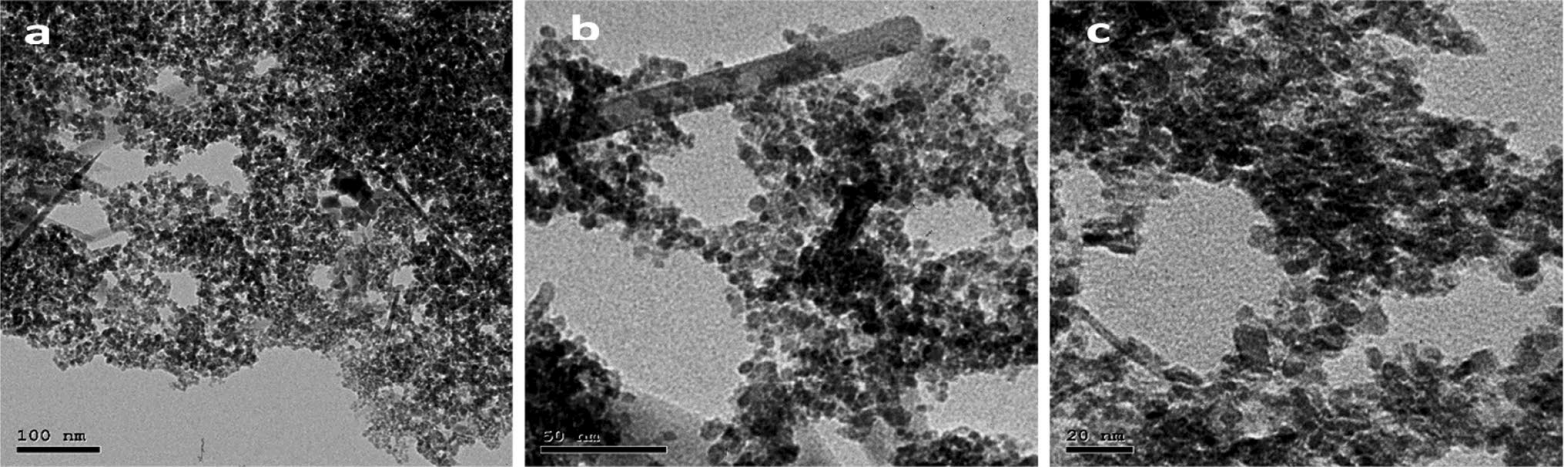
TEM images of PMC NPs with different magnifications (100, 50 and 20 nm)

TEM analysis clearly showed that the synthesized PMC NPs were nearly spherical with smooth surfaces, homogenously distributed without aggregation, and particle sizes ranging from 10 to 40 nm. Moreover, TEM showed different contrasts of PMC NPs which represent Fe_3_O_4_, chitosan, and piracetam (Fig. 2).

**Fig. 3:**
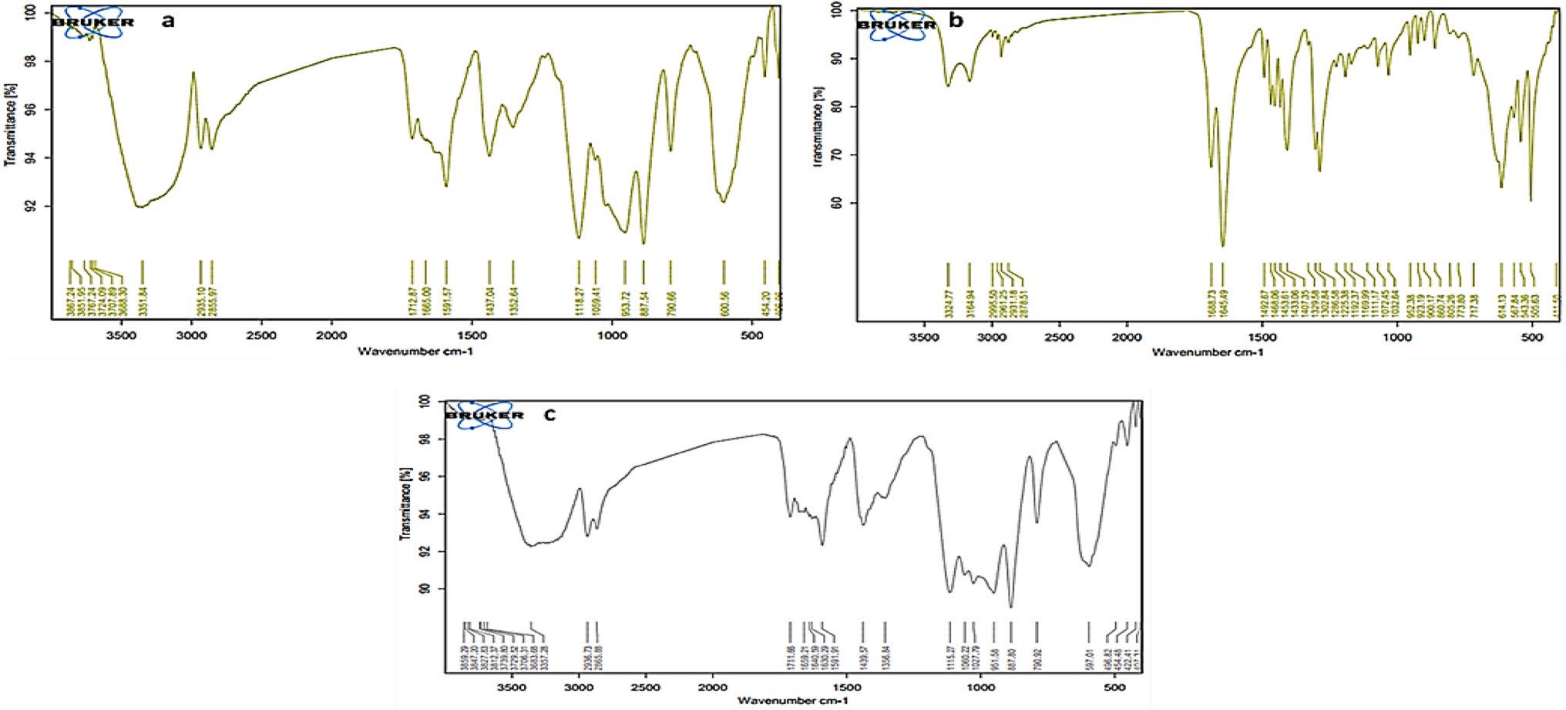
FTIR spectra of magnetic chitosan (**a**), piracetam (**b**), and PMC NPs (**c**)

According to FTIR spectroscopy (Fig. 3), piracetam showed its signature peaks at 1688 cm^−1^ (amide carbonyl group or NH_2_–O–C stretch), 1645 cm^−1^ (cyclic ketone group or C=O bands), 3164 and 3324 cm^−1^ (N–H stretching bands), and 1286 cm^−1^ (C–N stretching vibration). MC NPs showed their characteristic peaks at 3351 cm^−1^ (–OH stretching bands), 1665 cm^−1^ (–CO–NH_2_ stretching vibration), 1591 cm^−1^ (NH_2_ bending vibration), 1352 cm^−1^ (–C–O bending vibration), and 600 cm^−1^ is assigned to Fe–O group.

When piracetam was loaded on MC NPs, the peak’s location and shape were similar to that of MC NPs except for the absorption band at wave number 1665 cm^−1^ (stretching vibration of the amide groups of chitosan) was shifted to 1659 cm^−1^. Piracetam characteristic peaks at 1688 cm^−1^ (NH_2_–O–C stretch) shifted to 1640 cm^−1^ and the absorption band at 1645 cm^−1^ (C=O bands) shifted to 1630 cm − 1, which suggested that the amide groups of chitosan were linked to the carbonyl groups of piracetam in MC NPs.

### Encapsulation efficiency, loading capacity, and in vitro drug release.

Encapsulation efficiency (EE) and loading capacity (LC) of PMC NPs were calculated from the previous equations. The EE% and LC % were 81.2% and 36.1%, respectively.

**Fig.4:**
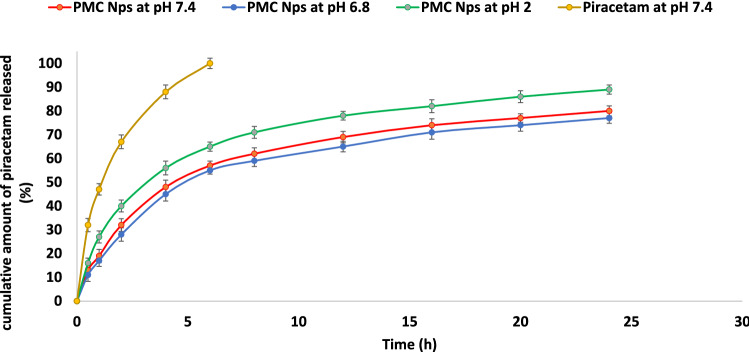
In vitro drug release profiles of the plain drug and PMC nanoparticles at different pH

The cumulative drug release percentage profiles of piracetam from PMC NPs in PBS (pH 7.4, 6.8, and 2, respectively) versus time can be observed in Fig. 3. It has come out that the release rate at acidic pH was faster than those in neutral or basic medium. As for the release profiles of piracetam at pH 7.4, PMC NPs displayed an initial rapid release of around 32% of piracetam in the first 2 h followed by a slow or sustained release profile resulting in the release of around 69% within 12 h. Correspondingly, the release profile of piracetam at pH 6.8 (Fig. 4) exhibited drug-releasing profiles similar to that at pH 7.4 with an immediate release profile in the first 2 h (around 28%) followed by slow release (around 65% within 12 h). However, the release profile at pH 2 (Fig. 4) showed a very fast or burst release profile with almost 40% of piracetam within 2 h of the experiment. After that, there was a relatively slower release profile (around 78% within 12 h).

### Assessment of behavioral neurotoxicity

#### Footprint analysis

**Fig. 5:**
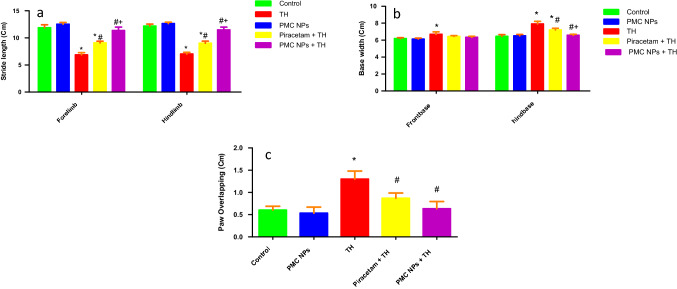
PMC NPs improved walking pattern of rats treated with TH. The walking pattern was assessed by footprint test in all groups including **a** fore and hind stride length, **b** front and hind base width, and **c** paw overlapping. TH treatment significantly shortened stride length by 42.1% and 42.5% for fore and hind stride length, respectively, while base width and paw overlapping were increased by 9.5%, 22.4% and 116.6% for front, hind base width, and paw overlapping, respectively, compared to control group which proved impaired walking pattern. Treatment with PMC NPs increased stride length by 65.4% and 63.7% for fore and hind stride length, respectively, and also reduced base width and paw overlapping by 5.2%, 16.9%, and 51.5% for front, hind base width, and paw overlapping, respectively, compared to TH-treated group that shows walking pattern improvement. Values are represented as mean ± SD (*n* = 6). Data were analyzed by one-way ANOVA followed by Tukey’s test. * symbolizes significant values as compared to controls (*P* < 0.01), # symbolizes significant values as compared to thiacloprid-treated animals (*P* < 0.01), and + symbolizes significant values as compared to piracetam-treated animals (*P* < 0.01)

PMC NPs improved the walking pattern of rats treated with TH as assessed by footprint test (Fig. 5). Marked effect of TH treatment was found on different walking pattern parameters including forelimb stride length [*F*(4, 25) = 145.857], hindlimb stride length [*F*(4, 25) = 233.842], front base width [*F*(4, 25) = 11.106], hind base width [*F*(4, 25) = 50.972], and paw overlapping [*F*(4, 25) = 29.433,] at *P* < 0.01. TH administration impaired walking pattern as manifested by considerably shortened stride length as compared to controls (*P* < 0.01). In addition, there was a notable increase in front and hind base width and paw overlapping in the TH-administered group as compared to control animals (*P* < 0.01). On the other hand, piracetam- and PMC NPs-treated rats showed an improved walking pattern with a significant increase in stride length of fore and hindlimbs as compared to the TH-treated group (*P* < 0.01). Moreover, base width and paw overlapping were significantly reduced in comparison to the TH-treated group (*P* < 0.01).

#### Hanging wire test

**Fig. 6:**
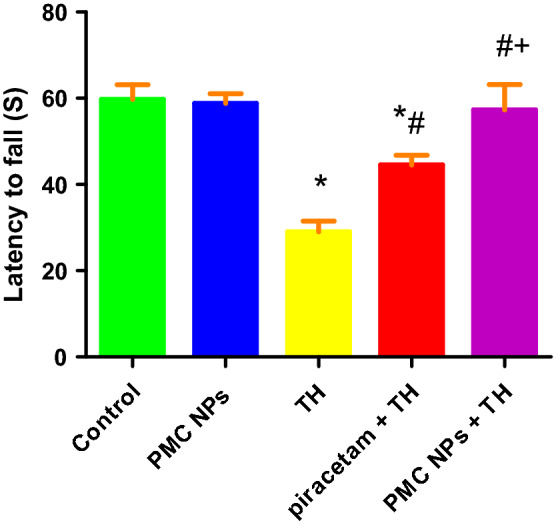
PMC NPs enhanced motor function and muscular strength of rats treated with TH. Motor function was assessed by hanging wire test in all groups in terms of latency to fall (s). TH treatment significantly reduced fall latency time (s) by 51.4% compared to control group which revealed impaired motor function and muscular strength. Treatment with PMC NPs increased fall latency time (s) by 97.7% compared to TH-treated group that displayed motor function and muscular strength enhancement. Values are displayed as mean ± SD (*n* = 6). Data were evaluated by one-way ANOVA followed by Tukey’s test. * symbolizes significant values in comparison to controls (*P* < 0.01), # symbolizes significant values in comparison to thiacloprid-treated animals (*P* < 0.01), + symbolizes significant values in comparison to piracetam-treated animals (*P* < 0.01)

Motor function and muscular strength were assessed following administration of piracetam and PMC NPs in treated rats with TH by hanging wire test in terms of latency to fall (Fig. 6). TH treatment showed a considerable reduction in fall latency [*F*(4, 25) = 83.972] at *P* < 0.01, indicating impaired muscular strength. Tukey’s post hoc test revealed a significantly reduced suspension time in TH-treated rats as compared to the control rats (*P* < 0.01). On the other hand, piracetam- and PMC NPs-treated rats exhibited a significant increase in the suspension time as compared to TH-treated rats (*P* < 0.01).

#### Open field test (OFT)

**Fig. 7:**
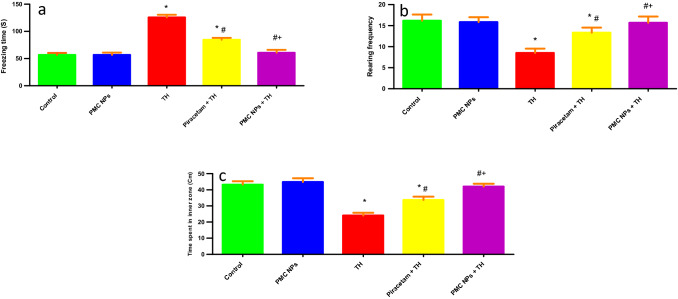
PMC NPs improved exploratory activity of rats treated with TH. Exploratory activity was assessed by open field test in all groups in terms of **a** freezing time (s), **b** rearing frequency, and **c** time spent in inner zone (s). TH treatment significantly increased freezing time (s) by 122.4% compared to control group while rearing frequency and time spent in inner zone (s) were markedly decreased by 47.4% and 44.4%, respectively, compared to control group which revealed anxiety, and behavioral despair. Treatment with PMC NPs decreased freezing time (s) by 51.6% compared to TH-treated group while rearing frequency time spent in inner zone (s) were notably increased by 84.2% and 75%, respectively, in comparison to TH-treated group that displayed enhanced exploratory activity. Data are displayed as mean ± SD (*n* = 6) and analyzed by one-way ANOVA followed by Tukey’s test. * symbolizes significant values as compared to controls (*P* < 0.01), # symbolizes significant values in contrast to thiacloprid-treated animals (*P* < 0.01), + symbolizes significant values in contrast to piracetam-treated animals (*P* < 0.01)

Exploratory activity was monitored through OFT following administration of piracetam and PMC NPs in TH-treated rats in terms of time spent in inner zone (s), freezing time (s), and rearing frequency (Fig. 7). Treatment with TH induced significant effects on time spent in the center squares [*F*(4, 25) = 110.344], the immobility period [*F*(4, 25) = 5279.417], and the rearing frequency [*F*(4, 25) = 37.181,] at *P* < 0.01 in OFT. TH-treated rats showed a notable decrease in time spent in the central squares (*P* < 0.01) and rearing frequency (*P* < 0.01) with a significant increase in the freezing time (*P* < 0.01) compared to control animals. However, in comparison to the TH group, piracetam- and PMC NPs-treated groups revealed a considerable increase in the time spent in central squares and rearing frequency (*P* < 0.01) with a decrease in the freezing time (*P* < 0.01).

#### Y-maze test

**Fig. 8:**
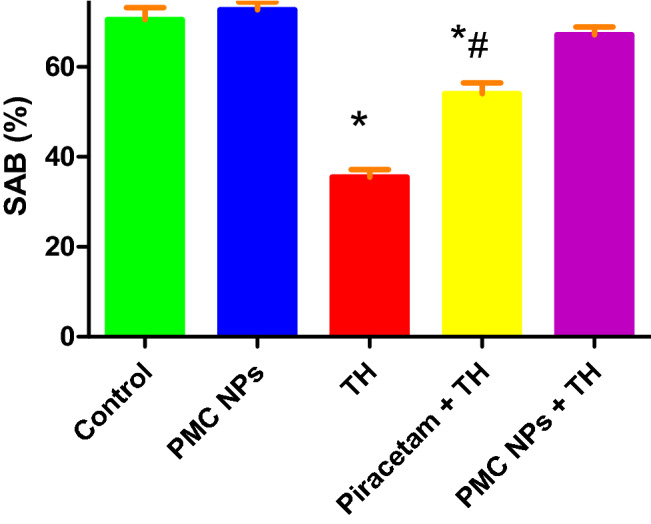
PMC NPs improved learning and memory impairment in rats induced by TH. Learning and memory functions were assessed by Y-maze test in all groups in terms of spontaneous alteration behavior (SAB) percentage. TH treatment significantly decreased SAB percentage by 49.6% compared to control group which revealed memory impairment. Treatment with PMC NPs significantly increased SAB percentage by 89.1% compared to TH-treated group that displayed improved learning and memory functions. Raw data are displayed as mean ± SD (*n* = 6) and analyzed via one-way ANOVA followed by Tukey’s test. * symbolizes significant values as opposed to controls (*P* < 0.01), # symbolizes significant values as opposed to thiacloprid-treated animals (*P* < 0.01), + symbolizes significant values as opposed to piracetam-treated animals (*P* < 0.01)

The effect of piracetam and PMC NPs on short-term working memory in TH-treated rats was assessed via the Y-maze test in terms of spontaneous alteration behavior (SAB) (Fig. 8). Y-maze test revealed that SAB was significantly affected by the given treatment [*F*(4, 25) = 329.18] at *P* < 0.01. TH-treated rats exhibited a substantially lower SAB in comparison to the control (*P* < 0.01). However, PMC NPs-treated rats revealed improved SAB as compared to TH- and piracetam-treated groups.

### Determination of AChE activity

**Fig. 9:**
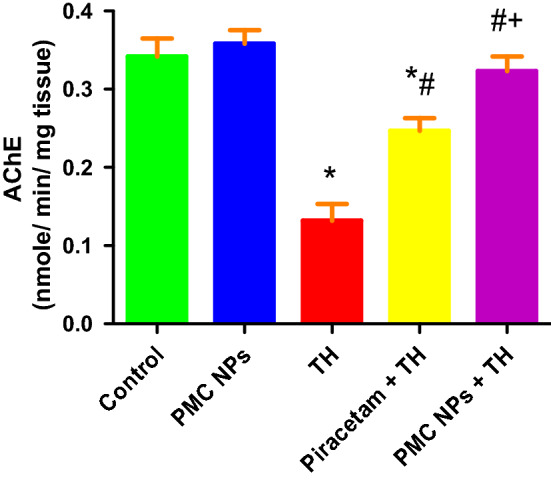
PMC NPs modulate AChE activity in brain tissue of rats treated with TH. TH treatment significantly decreased AChE activity by 61.5% compared to control group while treatment with PMC NPs markedly increased AChE activity by 145.5% compared to TH-treated group. Data are presented as mean ± SD (*n* = 6) and analyzed through one-way ANOVA followed by Tukey’s test. * symbolizes considerable values as opposed to controls (*P* < 0.01), # symbolizes considerable values as opposed to thiacloprid-treated animals (*P* < 0.01), + symbolizes considerable values as opposed to piracetam-treated animals (*P* < 0.01)

Effects of PMC NPs on brain AChE activity in rats were evaluated after exposure to TH (Fig. 9). Data showed a significant effect of TH treatment on AChE activity [*F*(4, 25) = 137.640] at *P* < 0.01 in the brain. TH treatment substantially reduced AChE activity when compared to the control group. However, both piracetam- and PMC NPs-treated groups significantly increased AChE activity as compared to the TH group (*P* < 0.01). Moreover, PMC NPs co-treated group significantly raised AChE activity as compared to the piracetam-treated group (*p* < 0.01).

### Determination of oxidative stress and antioxidant enzyme activities

**Fig. 10:**
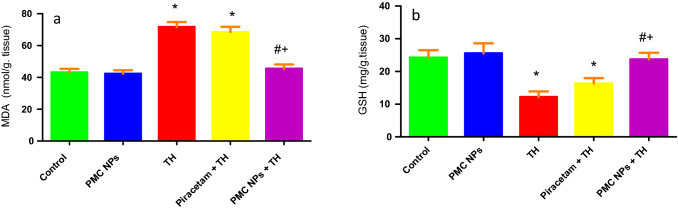
PMC NPs reduced oxidative stress in brain tissue of rats induced by TH. Oxidative stress was assessed by oxidative biomarkers in all groups in terms of **a** MDA **b** GSH. TH treatment significantly increased MDA by 66% compared to control group while GSH was markedly reduced by 49.7% compared to control group. Treatment with PMC NPs significantly decreased MDA by 36.5% compared to TH-treated group while GSH was significantly increased by 94.5% that displayed reduced oxidative damage. Data are represented as mean ± SD (*n* = 6) and analyzed via one-way ANOVA followed by Tukey’s test. * symbolizes considerable values in opposition to controls (*P* < 0.01), # symbolizes considerable values in opposition to thiacloprid-treated animals (*P* < 0.01), + symbolizes considerable values in opposition to piracetam-treated animals (*P* < 0.01)

**Fig. 11:**
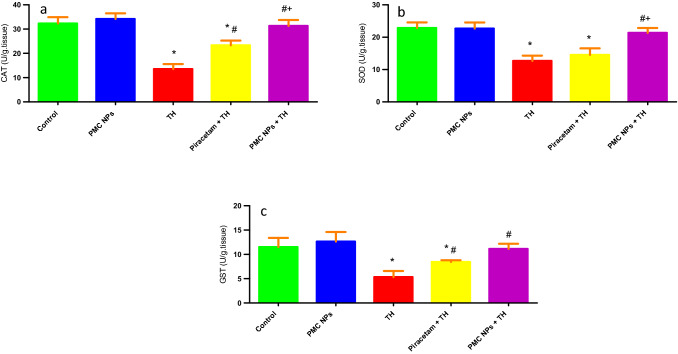
PMC NPs enhanced antioxidant capacity in brain tissue of rats treated with TH. Antioxidant capacity was assessed by antioxidant enzymes activity including **a** CAT, **b** SOD, and **c** GST. TH treatment significantly decreased CAT, SOD, and GST enzymes activity by 58.2%, 44.5%, and 53.7%, respectively, compared to control group. Treatment with PMC NPs significantly increased CAT, SOD, and GST enzymes activity by 132%, 68.5%, and 108.4%, respectively, compared to TH-treated group that revealed enhanced antioxidant capacity. Data are represented as mean ± SD (*n* = 6) and assayed via one-way ANOVA followed by Tukey’s test. * symbolizes considerable values in opposition to controls (*P* < 0.01), # symbolizes considerable values in opposition to thiacloprid-treated animals (*P* < 0.01), + symbolizes considerable values in opposition to piracetam-treated animals (*P* < 0.01)

GSH and MDA levels were determined in rat brain samples (Fig. 10). Data revealed a marked effect of TH treatment on GSH [*F*(4, 25) = 40.939] and MDA [*F*(4, 25) = 170.471] at *P* < 0.01. The TH-treated group showed a notable reduction in GSH (*P* < 0.01) and a significant rise in MDA (*P* < 0.01) as opposed to the control group. However, when compared to TH-treated rats, piracetam- and PMC NPs-administered groups significantly raised GSH levels (*P* < 0.01) and reduced MDA levels (*P* < 0.01). SOD, GST, and CAT activities were also assessed in brain tissue (Fig. 11). Data analysis revealed a remarkable effect of TH treatment on SOD [*F*(4, 25) = 44.878], GST [*F*(4, 25) = 25.601], and CAT [*F*(4, 25) = 84.664] at (*P* < 0.01) in opposition to control rats. However, both piracetam and PMC NPs co-treated groups displayed a significant rise in SOD, GST, and CAT activity in comparison to TH-treated group (*P* < 0.01).

### Assessment of proinflammatory cytokines

**Fig. 12:**
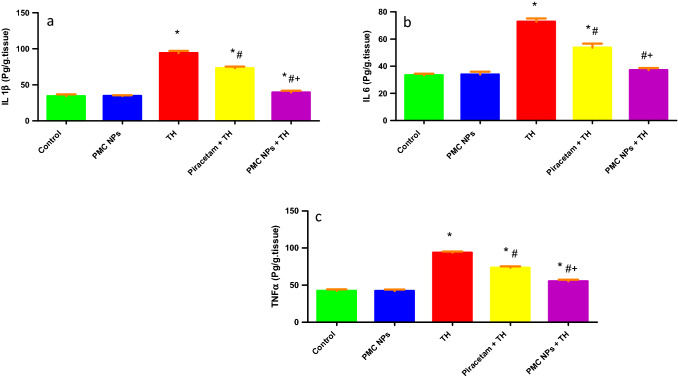
PMC NPs decreased proinflammatory cytokines in brain tissue of rats treated with TH. Proinflammatory cytokines levels including **a** IL1-β, **b** IL-6, and **c** TNF-α were evaluated. TH treatment significantly increased IL-1β, IL-6, and TNF-α levels by 173.8%, 118.3%, and 121.2%, respectively, compared to control group. However, treatment with PMC NPs significantly decreased IL-1β, IL-6, and TNF-α levels by 58%, 49%, and 41.1%, respectively, compared to TH-treated group that revealed enhanced anti-inflammation activity. Data are presented as mean ± SD (*n* = 6) and analyzed through one-way ANOVA followed by Tukey’s test. * symbolizes considerable values in comparison to controls (*P* < 0.01), # symbolizes considerable values in comparison to thiacloprid-treated animals (*P* < 0.01), + symbolizes considerable values in comparison to piracetam-treated animals (*P* < 0.01)

Proinflammatory cytokines IL-1β, IL-6, and TNF-α were evaluated in the brain tissue of rats (Fig. 12). Data revealed a considerable effect of TH treatment on cytokines levels including IL-1β [*F*(4, 25) = 780.617], IL-6 [*F*(4, 25) = 403.172], and TNF-α [*F*(4, 25) = 800.304] at *P* < 0.01. Tukey’s post hoc test showed that TH group revealed a notable increase in IL-1β, IL-6, and TNF-α (*P* < 0.01) as compared to the control group. However, both piracetam and PMC NPs co-treated groups showed a significant reduction in IL-1β, IL-6, and TNF-α in comparison to TH group (*P* < 0.01).

### Quantitative real-time PCR

**Fig. 13:**
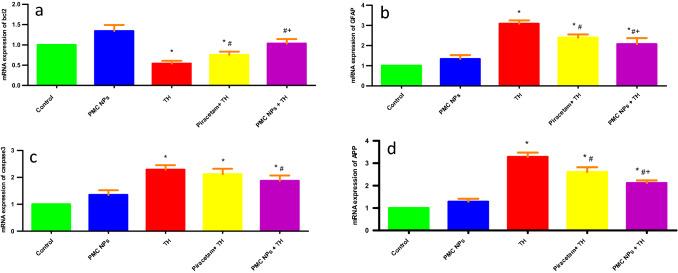
Genetic molecular analysis in brain tissue of rats following treatment with PMC NPs and TH. Relative mRNA expression level of **a** bcl2, **b** GFAP, **c** caspase-3, and **d** APP genes in brain tissue of rats was measured. TH treatment significantly decreased bcl2 relative mRNA expression level by 46.2% compared to control group while GFAP, caspase-3, and APP relative mRNA expression levels were markedly increased by 208.3%, 128.7%, and 228.3%, respectively, compared to control group. Treatment with PMC NPs significantly increased bcl2 relative mRNA expression level by 92.3% compared to TH-treated group while GFAP, caspase-3, and APP relative mRNA expression levels were decreased by 32.8%, 18.2%, and 35.6%, respectively, in comparison to TH-treated group. Data are displayed as mean ± SD (*n* = 6) and analyzed via one-way ANOVA followed by Tukey’s test. * symbolizes considerable values in contrast to controls (*P* < 0.01), # symbolizes considerable values in contrast to thiacloprid-treated animals (*P* < 0.01), + symbolizes considerable values in contrast to piracetam-treated animals (*P* < 0.01)

Relative mRNA expression levels of GFAP, APP, caspase-3, and BCL-2 genes in brain tissues of treated rats were conducted (Fig. 13). It was depicted that expressions of GFAP, APP, and caspase-3 genes were significantly upregulated in TH-intoxicated group in comparison to control group (*P* < 0.01). Meanwhile, these mRNA expressions were significantly downregulated after co-treatment with piracetam- and PMC NPs-treated group when compared to TH-treated group (*P* < 0.01).

In contrast, compared to the control group, relative mRNA expression of the anti-apoptotic Bcl-2 gene was significantly downregulated in TH-treated group (*P* < 0.01). This alteration could be reversed, and the bcl-2 gene was upregulated considerably after co-treatment with piracetam- and PMC NPs-treated group when compared to TH-treated group (*P* < 0.01). Moreover, PMC NPs group revealed the highest mRNA expression of bcl-2 gene.

### Histopathology and immunohistochemical examination

#### Cerebral cortex

**Fig. 14:**
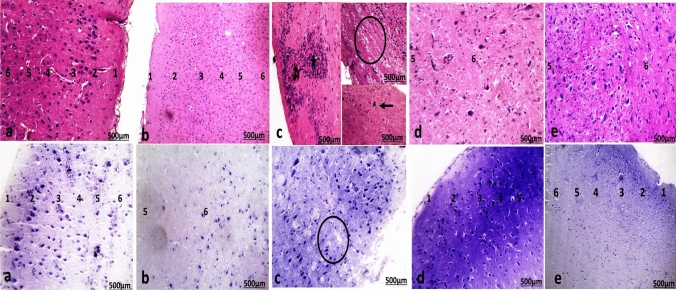
Photomicrographs of sections in the cerebral cortex of frontal lobe of **a** control rat showed well organized regularly arranged six layers from outer to inner surface: molecular layer (1), external granular (2), external pyramidal (3), internal granular (4), internal pyramidal (5), and polymorphic layer (6). The pia mater covers the molecular layer. **b** PMC NPs-treated rat showed well organized regularly arranged six layers. **c** Thiacloprid group revealed loss of organization of layers and the molecular layer revealed deformed neurons surrounded by haloes (circle) and infiltration of inflammatory cells (*).**d** Piracetam co-treated with thiacloprid group had deeply stained nuclei and surrounded by haloes. The pyramidal cells were deformed with deeply stained nuclei and surrounded by haloes. **e** PMC NPs co-treated with thiacloprid group showed normal cerebral cortex architecture with its normal arrangement of their layers. (H.&E. stain and crystal violet stain ×100)

Examination of cerebral sections of control rat and PMC NPs groups showed the same results (Fig. 14a) and (Fig. 14b). Sections revealed six layers of gray matter that are regularly arranged from outer to inner into, outer molecular, external granular, external pyramidal cell, internal granular, internal pyramidal, and lastly polymorphic cell layer.

Examination of cerebral sections of rats treated with TH revealed a loss of layers organization (Fig. 14c). The molecular layer revealed deformed neurons surrounded by haloes. The neuropil was vacuolated. In the internal pyramidal layer, deformed neurons appeared with deeply stained nuclei and were surrounded by haloes. The cerebral cortex showed infiltration of inflammatory cells.

The cerebral cortex of piracetam co-treated with TH group had deeply stained nuclei and was surrounded by haloes. The pyramidal cells were deformed with deeply stained nuclei (Fig. 14d).

Cerebral sections of PMC NPs co-treated with TH group showed cerebral cortex with normal histological structure and architecture with the nearly normal arrangement of its layers (Fig. 14e).

#### Hippocampus

**Fig. 15:**
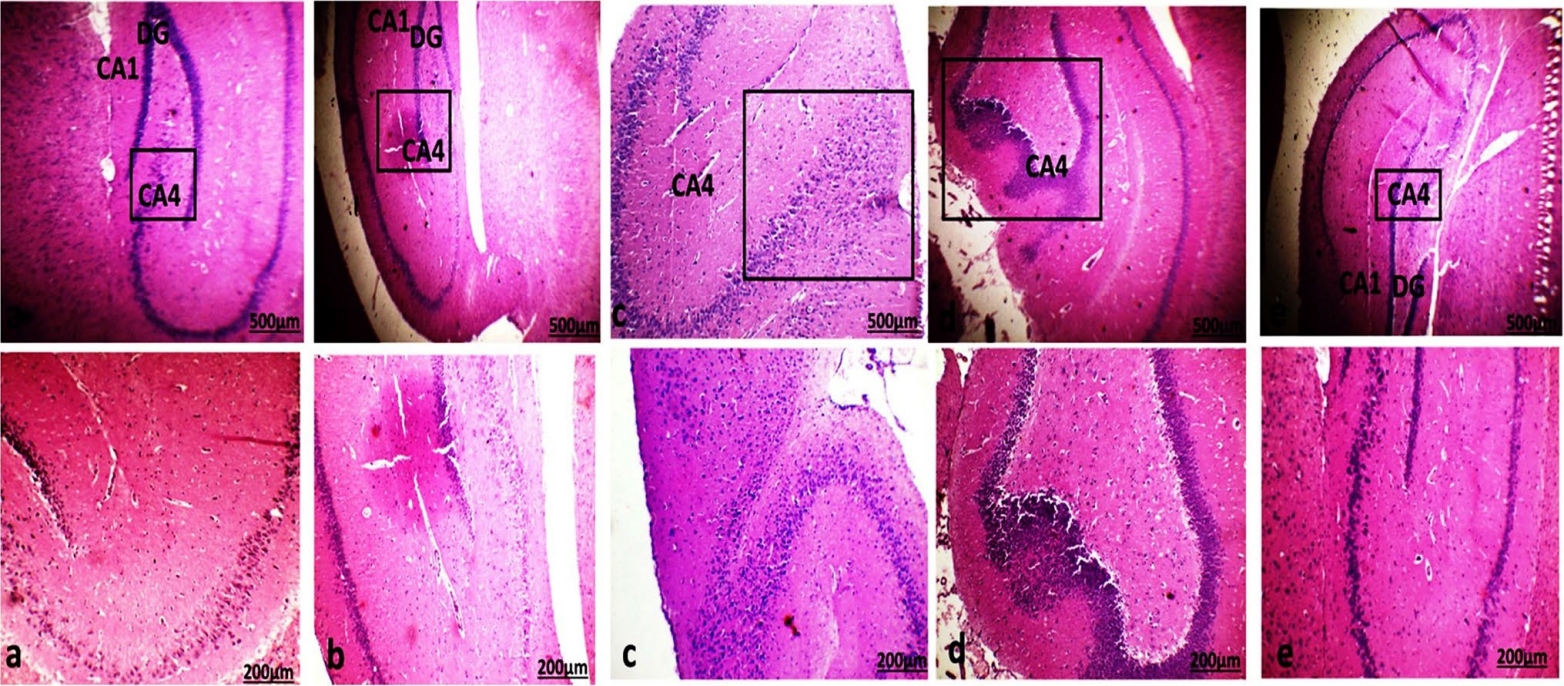
Photomicrographs of sections in the hippocampus sections and magnified part (rectangle) of **a** control rat **b** PMC NPs-treated rat showed normal histological structure. The hippocampus appeared as C-shaped Cornu Ammonis (CA1, CA2, CA3, and CA4), dentate gyrus, and subiculum. **c** Thiacloprid group and **d** piracetam-co-treated thiacloprid group revealed extravasation of the inflammatory cells in the parenchyma of Cornu Ammonis CA4 square. **e** PMC NPs co-treated with thiacloprid rats showed normal histological structure. (*H.&E.* stain ×100 and ×200)

Examination of hippocampus sections of control and PMC NPs groups showed normal histological structure (Fig. 15). The hippocampal was properly subdivided into four distinct regions (CA1, CA2, CA3, and CA4) according to its major constituent pyramidal cells. Examination of hippocampal sections of TH group and piracetam co-treated with TH group revealed extravasation of the inflammatory cells in the parenchyma of Cornu Ammonis CA4 (Fig. 15c) and (Fig. 15d). Examination of hippocampal sections of PMC NPs co-treated with TH group revealed normal histological structure (Fig. 15e).

#### Sciatic nerve

**Fig. 16:**
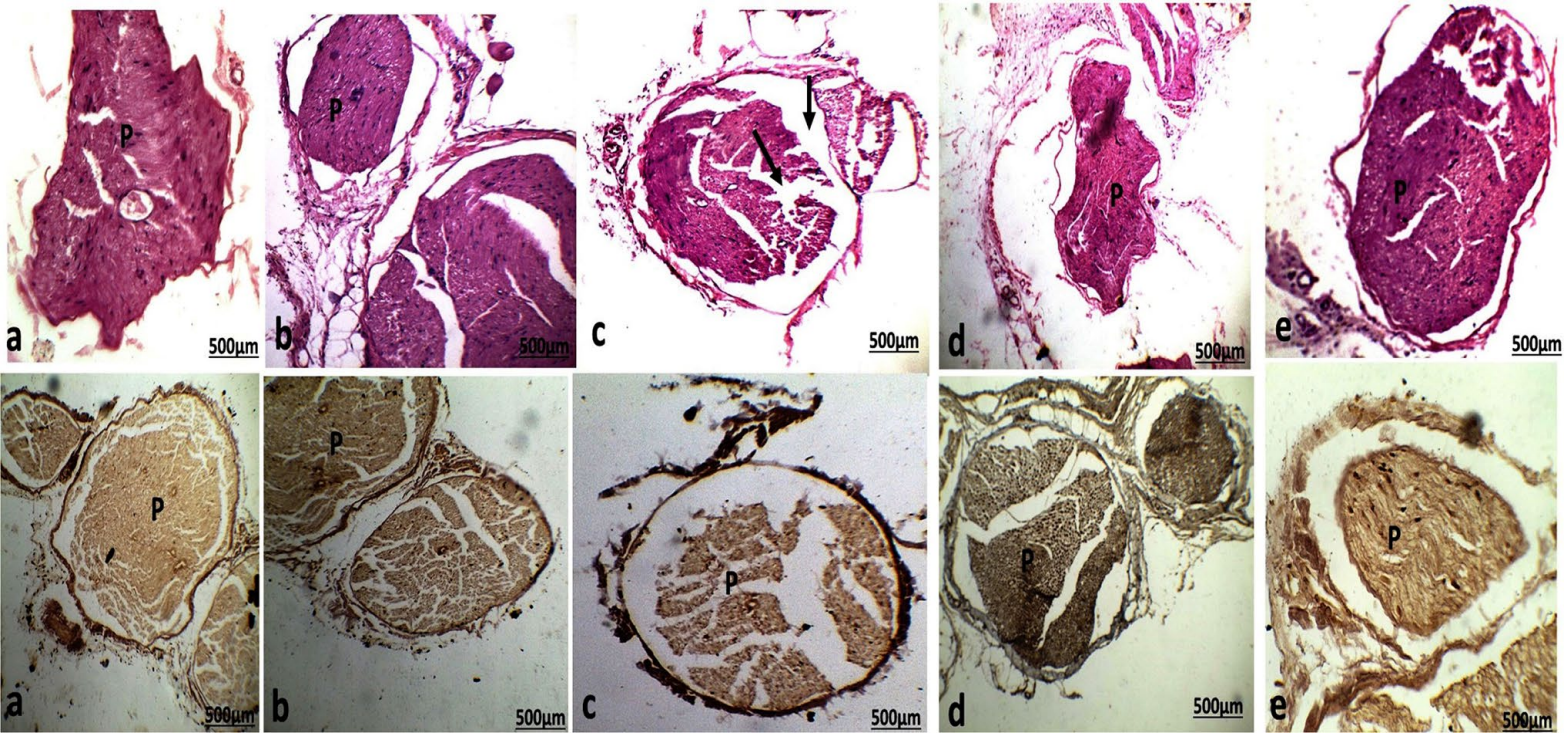
Photomicrographs of sagittal sections in the sciatic nerve of **a** control rat **b** PMC NPs-treated rat showed normal histological structure of the bundle of nerve fibers (*p*). **c** Thiacloprid group, the bundle of nerve fibers tended to separate from each other, and a space and degenerated fibers were clearly observed (arrow). **d** Piracetam-co-treated thiacloprid group and **e** PMC NPs-co-treated thiacloprid rats showed normal strong adherence nerve fibers (*P*). (*H&E.* stain and silver nitrate stain ×100

Examination of sciatic nerve sections of control and PMC NPs-treated groups showed a normal histological structure with strong adherence between the nerve fibers (Fig. 16). In TH group, nerve fibers bundles tended to separate from each other and degenerated fibers (myelin and Schwann cells) were observed (Fig. 16c). Conversely, piracetam-co-treated TH group and PMC NPs-co-treated TH groups showed strong adherence and thickening of nerve fibers bundles (Fig. 16d) and (Fig. 16e).

#### Immunohistochemical expression of tau protein in the cerebral cortex and cerebellum

**Fig. 17:**
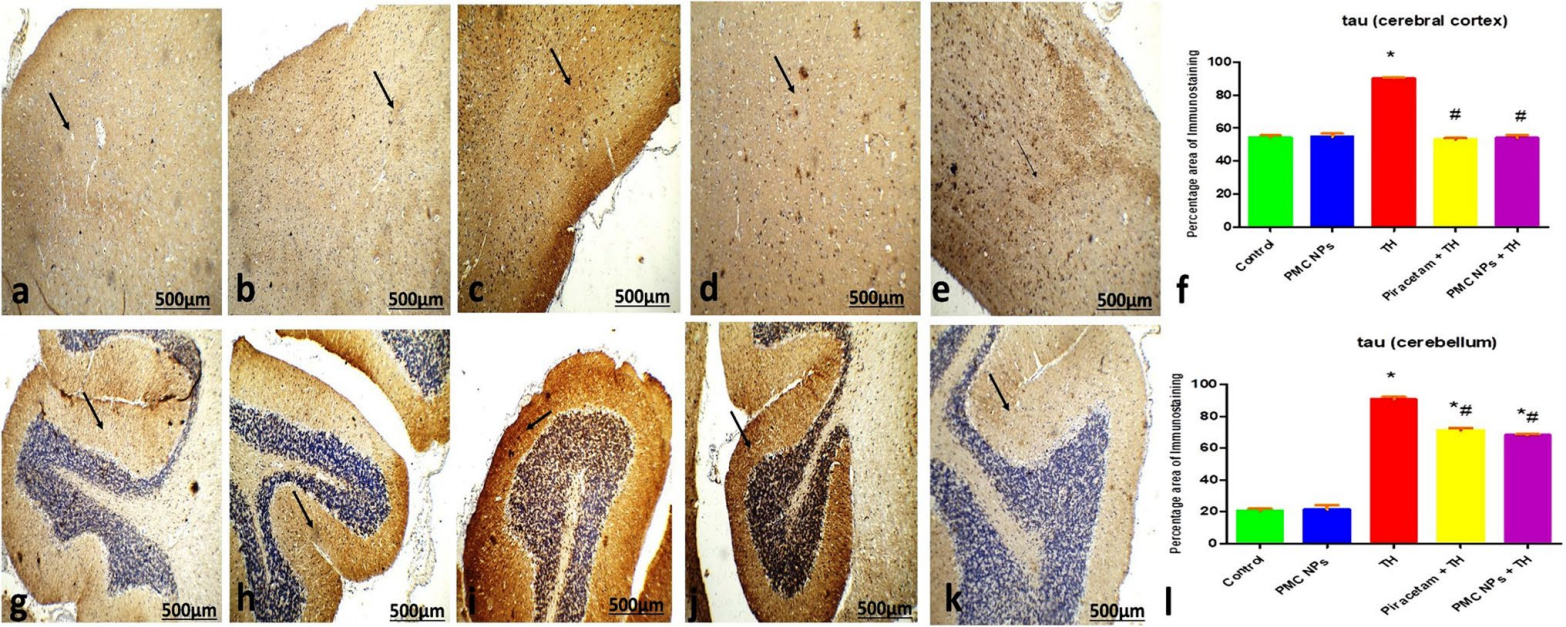
Photomicrographs of sections in the cerebral cortex and cerebellum sections of **a** and **g** control rat **b** and **h** PMC NPs-treated rat showed scattered tau immunoreaction of the nerve cells. **c** and **l** Thiacloprid group showed increase positive tau immunoreaction of the nerve cells. **d** and **j** Piracetam co-treated with thiacloprid and **e** and **k** PMC NPs co-treated with thiacloprid groups showed scattered tau immunoreaction of the nerve cells. (tau immunohistochemical stain ×100). **f** and **i** Histogram of the percentage area of immunohistochemical expression of tau between different group. Values are represented as mean ± SE. Data were analyzed by one-way ANOVA followed by Tukey’s test. * represents significant values when compared to controls at *P* < 0.01, # represents significant values when compared to thiacloprid-treated animals at *P* < 0.01, + represents significant values when compared to piracetam-treated animals at *P* < 0.01

Examination of the cerebral cortex and cerebellum sections of control and PMC NPs groups showed significantly decreased tau protein immunoreaction of the nerve cells and their cellular layers shown in Fig. 17a and g and Fig. 17b and h, respectively.

The cerebral cortex and cerebellum sections of TH group showed significantly increased tau protein immunoreaction of the nerve cells and their cellular layers compared to the control group (Fig. 17c and i).

Examination of the cerebral cortex and cerebellum sections of piracetam co-treated with TH and PMC NPs co-treated with TH groups revealed a normal decrease of tau immunoreaction of the nerve cells and their cellular layers compared to TH group, shown in Fig. 17d and j and Fig. 17e and k, respectively.

#### Immunohistochemical expression of NF-κB in sciatic nerve

**Fig. 18:**
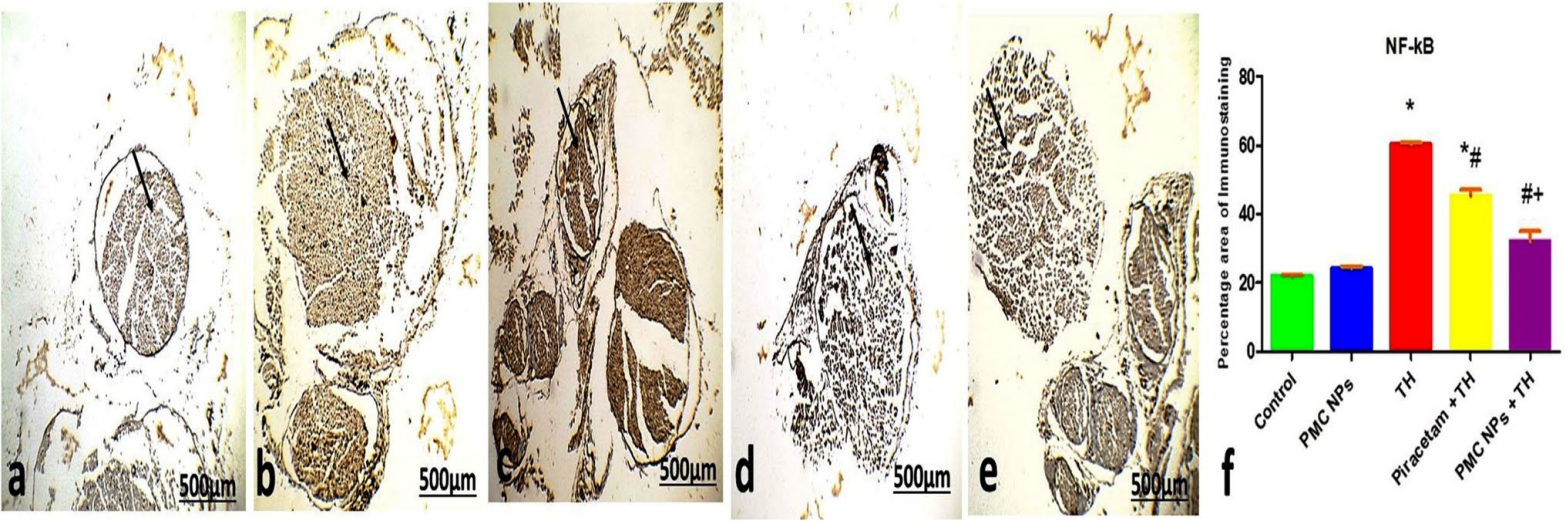
Photomicrographs of sections in the sciatic sections of **a** control rat **b** PMC NPs-treated rat showed scattered NF-κB immunoreaction of the bundle cells. **c** Thiacloprid group showed increase positive NF-κB immunoreaction of the bundle cells. **d** Piracetam co-treated with thiacloprid group and **e** PMC NPs co-treated with thiacloprid rats showed scattered NF-κB immunoreaction of the bundle cells. (NF-κB immunohistochemical stain ×100). **f** Histogram of the percentage area of immunohistochemical expression of NF-κB between different group. Values are represented as mean ± SE. Data were analyzed by one-way ANOVA followed by Tukey’s test. * represents significant values when compared to controls at *P* < 0.01, # represents significant values when compared to thiacloprid-treated animals at *P* < 0.01, + represents significant values when compared to piracetam-treated animals at *P* < 0.01

Examination of sciatic sections of control rat and PMC NPs-treated groups showed significantly decreased NF-κB immunoreaction in the bundle of the sciatic nerve, shown in Fig. 18a and Fig. 18b, respectively. While sciatic sections of TH group showed significantly increased positive immunoreaction for NF-κB (Fig. 18c) compared with the control group. Piracetam co-treated with TH and PMC NPs co-treated with TH showed less prominent and weak NF-κB immunohistochemical staining compared with TH group and nearly similar to the control group, shown in Fig. 18d and Fig. 18e, respectively.

## Discussion

In this study, PMC NPs were successfully synthesized using co-precipitation followed by an ionic gelation technique, piracetam was encapsulated in MC NPs. TEM was used to evaluate the uniformity of shape, size, and physical stability and revealed that PMC NPs attained typical spherical core–shell structure with the darker Fe_3_O_4_ nanoparticle seen as the core while the gray-colored layer surrounding it representing the chitosan polymer and piracetam which is agreed with previous reports (Rathinam et al. 2021; Zamora-Mora et al. 2014).

Consistently, FTIR is considered one of the most precise devices for investigating chemical structure and confirming the presence of functional groups on a synthesized nanoparticle's surface (Reddy and Lee 2013). Our results exhibited that piracetam was loaded efficiently onto MC NPs since the characteristic peaks of piracetam at 1688 cm^−1^ (NH_2_–O–C stretch) and 1645 cm − 1 (C=O bands) shifted to 1640 cm^−1^ and 1630 cm^−1^, respectively, besides the appearance of the new absorption peak at 1659 cm^−1^; these results go along with previous reports which suggest competent bonding between piracetam and MC NPs that may be through the amide and carbonyl functional groups of chitosan and piracetam, respectively (Gambhire et al. 2021; Li et al. 2020).

Encapsulation efficiency and loading capacity are crucial factors that explain the reasonability of MC NPs formulation (Dong et al. 2017). The encapsulation efficiency is determined as the percentage of piracetam entrapped in the magnetic chitosan nanocarrier (Amiri et al. 2021). Our data revealed EE% was 81.2% which implied a good affinity and is in harmony with earlier reports (Gooneh-Farahani et al. 2020; Taherian et al. 2021). On the other hand, LC% which measures the amount of piracetam associated with nanoparticles based on the weight of the magnetic chitosan nano-polymer exhibited a relative decrease (36.1%) that may be due to the occupation of some chitosan active sites by magnetite nanoparticles forming the core–shell structure (Rajesh et al. 2021). Similar findings have been recorded with isoniazid in MC NPs (Qin et al. 2015).

In vitro release profile of piracetam from PMC NPs displayed a typical biphasic release process with an initial burst or fast release in the early hours followed by a slow release rate subsequently, since rapid initial piracetam release may be due to drug molecules located on the surface of the PMC NPs while sustained release profile was related probably to the drug molecules trapped or diffused inside the nanoparticles (Eskandani et al. 2022; Pechanova et al. 2019). The in vitro release profile also revealed that the drug at various pH conditions was very stable whereas the fast release profile at pH 2 compared to the basic or neutral medium can be justified because, at this pH, the MC NPs acquired a positive charge with protonation of the amino groups in chitosan which resulted in weaker piracetam–magnetic chitosan interaction (An and Dultz 2007; Rajesh et al. 2021). These results coincide with previous studies which revealed that the physicochemical properties of nanoparticles could affect the release profile of drugs (Eskandani et al. 2022; Gaihre et al. 2009).

The current study is designed to evaluate the effect of PMC NPs against TH-induced neurotoxicity in albino rats. The most striking results of this study are that TH-induced oxidative brain injuries with subsequent elevation of proinflammatory cytokines potentially provoked cellular and molecular neurodegenerative changes in brain tissue. These chain events resulted in neurobehavioral deficits in TH-treated rats. However, piracetam and PMC NPs reversed most of those events, PMC NPs resulted in more notable improvement than piracetam alone.

Naughton et al. (2017) suggested that pesticides including TH evoked some psychiatric disorders such as motor dysfunction, depression or anxiety (Harrison and Ross 2016), and cognitive impairment (Ramírez-Santana et al. 2020). Consistent with the previous studies, our results revealed that TH administration impaired locomotor activity and gait performance besides reduced muscular strength that may be attributed to peripheral neuropathy and neuromuscular transmission blockage (Anadón et al. 2020; Farag et al. 2021; Haider et al. 2020; Khalil et al. 2017). However, PMC NPs significantly improved motor deficits induced by TH. These findings coincided with Fayez et al. (2019) who reported that oral treatment of albino rats with piracetam (600 mg/kg Bwt.) improved synaptic transmission in vascular dementia induced by l-methionine.

OFT was applied for the assessment of exploration and anxiety-related behavior (Khalil et al. 2017). Animals exposed to TH displayed an increase in the freezing time together with the reduction in the central squares' time and rearing behaviors suggesting that TH could elicit a stress response in rats (Bharatiya et al. 2020) that may be attributed to the disruption of some brain structures as the basolateral amygdala and hippocampus with functional impairment (Severyukhin et al. 2021). These changes were significantly reversed in PMC NPs-treated group. Our data are similar to Tripathi et al. (2017) that revealed that intraperitoneal injection of piracetam (200 mg/kg Bwt.) reversed LPS-induced cognitive impairment in albino rats.

Cognitive changes have been associated with exposure to several pesticides (Kara et al. 2015); the current study revealed that TH administration induced cognitive impairment suggesting weakened spatial memory. These results coincided with Mora-Gutiérrez et al. (2021) who reported that subchronic oral administration of TH (160 mg/kg Bwt.) every 72 h for 27 days could elicit impairment in short-term memory in rats. PMC NPs resulted in more prominent improvement in the spatial working memory than piracetam which might be due to amendment of AChE activity and cholinergic functions, neuronal cell damage inhibition, increasing cerebral blood flow and metabolism, and antioxidant activity (Malykh and Sadaie 2010; Moran et al. 2002; Wojszel 2021).

AChE catalyzes the hydrolysis of the acetylcholine neurotransmitter and terminates nerve impulses, so it is considered the target site of inhibition by several pesticides (Stara et al. 2021). Our findings displayed that TH administration drastically decreases the AChE activity, which is in line with the previous studies (Farag et al. 2022; Stara et al. 2021; Uçkun and Özmen 2021). Inhibition of AChE indicated that TH causes toxic effects related to the cholinergic system (Uçkun and Özmen 2021) which causes impairment of cognitive and motor functions (Hasan et al. 2020). On the other hand, PMC NPs significantly reversed these changes through the restoration of AChE activity. Similarly, Abdel-Salam et al. (2011) displayed that intraperitoneal injection of piracetam (300 mg/kg Bwt.) could enhance AChE activity and cholinergic function in ethidium bromide-induced acute demyelination of rats' brain.

Reactive oxygen species threaten neuronal survival and initiate an oxidative chain reaction damaging various areas of the brain; hence, the progression of cognitive deficits and dementia is influenced by oxidative stress (Hussein et al. 2018) since oxidative stress could trigger an intracellular proinflammatory signaling cascade with activation of acute phase inflammatory responses (Aboubakr et al. 2021). The current study revealed that TH triggered a marked decrease in the activity of the antioxidant enzymes with a significant elevation of MDA level that may be due to the accumulation of reactive oxygen species (ROS) and free radicals which could exhaust the antioxidant defense system, these results coincided with previous studies as Hendawi et al. (2016) who reported that rats exposed to TH at dose level 22.5 mg/kg Bwt. orally for 30 successive days showed a significant decrease in GST, while MDA significantly reduced. Concurrent treatment with piracetam and PMC NPs alleviated TH-induced oxidative stress at variable levels. Furthermore, PMC NPs have a more prominent scavenging capacity than piracetam alone. A previous study reported that piracetam at a dose level 300 mg/kg Bwt. administered intraperitoneally daily for 45 days could reverse oxidative damage induced by AlCl_3_ in rats’ brain tissue (Abdel-Salam et al. 2016). Moreover, Kalkan et al. (2011) revealed that piracetam at a dose level of 250 mg/kg Bwt. suppressed MDA in rabbit's spinal cord ischemia reperfusion injury; however, Horvath et al. (2002) reported that at therapeutic concentrations, piracetam had negligible antioxidant activity and showed scavenging capacity only at 10-times higher concentrations than the therapeutic concentrations. Hence, our results could explain the added apparent value of MC NPs in increasing the antioxidant efficacy of piracetam as reported previously (Feyzioglu and Tornuk 2016; Ou et al. 2022; Silva et al. 2020). Moreover, the formulation of piracetam in nanoparticulate forms exhibited better therapeutic efficacy contrary to piracetam alone in terms of antioxidant, anti-apoptotic, and anti-inflammatory activities (Nasr and Wahdan 2019), hence delineating such formulation as an innovative promising technique of brain nootropics drug delivery.

The proinflammatory cytokines are synthesized and released by both activated microglia and astrocytes in brain tissue and establish a connection between specific and innate immunity (Wang et al. 2015). Increased concentrations of these cytokines are suggested to be the initial events in the emergence of neurodegenerative diseases (Gargouri et al. 2018). In this study, TNF-α, IL-1β, and IL-6 were significantly increased in the rat brains after exposure to TH. This finding is in line with Xie et al. (2022) who revealed that treatment with waterborne TH (40 μM) in zebrafish for 21 days significantly elevated TNF-α and IL-6 levels by 2.5-fold. Furthermore, the present investigation exhibited that PMC NPs mitigated TH-induced inflammation more effectively than piracetam alone with a marked reduction in TNF-α, IL-1β, and IL-6 levels in the TH-exposed rats. This agrees with other reports emphasizing that high doses of piracetam may enhance the alleviation of chemically induced inflammatory responses (Abdel-Salam et al. 2016; Navarro et al. 2013; Verma et al. 2018); hence, MC NPs as reported previously could improve drug efficacy with lower doses of drugs and enhance its anti-inflammatory effects (Kim 2018; Rostami 2020).

For further understanding of the molecular mechanism of TH neurotoxicity and the protective effect of PMC NPs, gene expression profiles of GFAP, APP, caspase 3, and Bcl-2 were performed in the brain tissue of rats. GFAP is an intermediate filament protein of astrocytes, considered a specific marker of astrocyte activation and/or injury that usually occur following exposure to neurotoxic elements or other neurologic disorders (Gust et al. 2022; Yang et al. 2022). The present report showed a significant upregulation of the GFAP gene in TH-intoxicated group. This may be due to the elevation of oxidative stress and excessive generation of free radicals since astrocytes that maintain the integrity of the blood–brain barrier are considered a target of oxidative damage (Carvajal-Flores et al. 2020; Fulton et al. 2021). These findings lie in the same line with previous reports that declared the elevated expressions of GFAP were assumed to be a sensitive marker of neurotoxins such as TH (Forner-Piquer et al. 2021; Martínez-Larrañaga 2020).

Correspondingly, TH exhibited a significant elevation of APP gene expression in brain tissues. APP is a type of transmembrane glycoprotein that plays an important role in neuronal homeostasis, synaptic plasticity, and cell adhesion (Chen et al. 2017). It is worth mentioning that APP sequential processing via secreting enzymes triggered the release of beta-amyloid into the CNS extracellular space while dysregulated levels of APP and its catabolites lead to the accumulation of insoluble amyloid plagues which trigger the production of ROS, neural cell death, and various neurological disorders including Alzheimer's disease (Westmark 2013; Zetterberg et al. 2010). Recently, insecticides such as TH have been extensively linked to neuropathology since oxidative stress, neuroinflammation, and specific Aβ- and tau-related pathways could be involved in the neuropathological mechanisms (Tang 2020).

It is well known that apoptosis is generally mediated through activation of either the extrinsic or intrinsic (mitochondrial) pathways so mitochondrial membrane potential change could induce apoptosis in committed cells through oxidative stress since the increased level of ROS induces signaling responsive cascades that activate transcription of various genes and ultimately lead to apoptosis (Quast et al. 2013; Sarkar et al. 2011). The present study revealed that TH significantly increased the expression of the pro-apoptotic caspase-3 that can promote the signal transduction way of apoptosis and activate DNA damage through the improvement of caspase-activated deoxyribonuclease in TH-intoxicated group (Alam et al. 2019). On contrary, TH displayed a marked downregulation in the expression of anti-apoptotic Bcl-2 marker as a response to the oxidative stress evoked by TH since Bcl-2 has a significant impact on encouraging cell survival, regulating the antioxidant pathway, and preventing pro-apoptotic protein actions to avoid apoptosis and cell damage (Zimmermann et al. 2001). These findings are consolidated with Schwarzbacherová et al. (2019) who displayed that exposure to TH at 240 μg/ml for 4 h could provoke oxidative damage and apoptosis in bovine peripheral lymphocytes.

On the other hand, the current study presented that elevated expression levels of GFAP, APP, and caspase-3 were significantly decreased meanwhile the expression of Bcl2 was enhanced following co-treatment with piracetam and PMC NPs so their roles in counteracting the neurotoxicity of TH cannot be ignored. These findings lie in the same line with previous studies and the above-mentioned data since piracetam displays improved mitochondrial functions via enhancing mitochondrial membrane fluidity, also piracetam mediated a cytoprotective activity and prevents the astrocyte's apoptosis (Gabryel et al. 2002; Kurz et al. 2010). Furthermore, the protective effects of piracetam could be attributed to its nature in modulating the hippocampal cholinergic receptors besides inhibition of the lipid-destabilizing effect of the amyloid peptide and enhancement of memory deficit (Mingeot-Leclercq et al. 2003; Stahlhut et al. 2014). Moreover, piracetam could attenuate oxidative stress-mediated neurodegeneration via the reduction of proinflammatory cytokines and downregulation of GFAP and caspase-3 expression in brain tissue (Verma et al. 2018). Interestingly, PMC NPs beneficial effects exceed that of piracetam alone which might be due to the antioxidant and anti-inflammatory effect of PMC NPs.

The current study also examined the histopathological and immunohistochemical alterations in brain tissue (cerebral cortex, hippocampus, and cerebellum) and sciatic nerve of rats to emphasize the protective effect of PMC NPs against TH-induced neurotoxicity. The cerebral cortex of TH-treated rats revealed marked disorganization, infiltration of inflammatory cells, and damage of cortical layers, also pyramidal and granular cells lost their normal shape and were surrounded by haloes.

Moreover, TH-treated animals exhibited visible unstained haloes surrounding the Purkinje cell with vacuolation in the nearby cerebellar molecular and granular layers owing to the contraction of Purkinje cells and loss of protoplasmic processes (Abdel-aziz et al. 2019). These results coincide with Ghoneim et al. (2014) who reported vacuolization in the brain cells due to exposure to free radicals that were released from tissue damage and destroyed cell organelles.

Piracetam and PMC NPs showed improvement in the histological structure of the brain tissue (cerebral cortex, cerebellum, and hippocampus) and the sciatic nerve which could be due to the antioxidant effect of piracetam and PMC NPs revealed in our study. Furthermore, Solanki et al. (2011) demonstrated that piracetam (1 mM) significantly decreased ROS generation and prevented oxidative membrane damage in primary hippocampal culture following hypoxia-reoxygenation-induced injury.

Immunohistochemical analysis of TH-treated rats interestingly showed a significant increase in the percentage area of tau expression in the cerebral cortex and cerebellum that could play a crucial role in the cognitive deficits via elevation of neurofibrillary tangles (NFTs) number (Shan et al. 2016). Moreover, a notable increase in the immunohistochemical expression of NF-κB in the sciatic nerve caused a profound release of proinflammatory cytokines (Sakai et al. 2000). On the other hand, piracetam and PMC NPs showed a marked decrease in the percentage area of immunohistochemical tau protein expression in the cerebral cortex and cerebellum and NF-κB expression in the sciatic nerve that may be explained by its capacity to reduce oxidative stress as shown in the present study which in turn supporting the neuroprotective effect of PMC NPs against TH-induced neurotoxicity.

## Conclusion

TH exposure leads to oxidative stress and inflammatory and neurobehavioral deficits with marked degenerative and histopathological changes in the brain tissue and sciatic nerve. Furthermore, TH could induce neuronal apoptosis and provoke disruption of neuronal homeostasis via GFAP/APP pathway, and these findings suggested that multiple integrated mechanisms are involved in TH-induced neurotoxicity. These changes were regressed to a lesser extent when using PMC NPs with TH in comparison with piracetam alone since treatment with PMC NPs exerts an anti-inflammatory, antioxidative, and anti-apoptotic effect in addition to modulating of GFAP/APP pathway and restoring synaptic plasticity that may be responsible for the amelioration of TH neurotoxicity. PMC NPs may be recommended as a promising novel approach for remedying neurological diseases and modulating the neurogenic niche and neuroinflammatory environment.

## Data Availability

Data supporting findings are presented within the manuscript.
